# COVID-19 vaccine testing & administration guidance for allergists/immunologists from the Canadian Society of Allergy and Clinical Immunology (CSACI)

**DOI:** 10.1186/s13223-021-00529-2

**Published:** 2021-03-15

**Authors:** Timothy K. Vander Leek, Edmond S. Chan, Lori Connors, Beata Derfalvi, Anne K. Ellis, Julia E. M. Upton, Elissa M. Abrams

**Affiliations:** 1 Pediatric Allergy and Asthma, Department of Pediatrics, University of Alberta, 207-10430 61 Ave NW, Edmonton, AB T6H 2J3 Canada; 2 Division of Allergy and Immunology, Department of Pediatrics, University of British Columbia, BC Children’s Hospital, Vancouver, BC Canada; 3 Department of Medicine, Dalhousie University, Halifax, NS Canada; 4 Division of Immunology, Department of Pediatrics, Dalhousie University/IWK Health Centre, Halifax, NS Canada; 5 Division of Allergy & Immunology, Department of Medicine, Queen’s University, Kingston, ON Canada; 6 Division of Immunology and Allergy, Hospital for Sick Children, Department of Paediatrics, University of Toronto, Toronto, ON Canada; 7 Department of Pediatrics, Section of Allergy and Clinical Immunology, University of Manitoba, Winnipeg, MN Canada; 8 Department of Pediatrics, Division of Allergy and Immunology, University of British Columbia, Vancouver, BC Canada

**Keywords:** COVID-19, Vaccine, PEG, Allergy, Anaphylaxis, Immunocompromise, Immune deficiency

## Abstract

**Background:**

Safe and effective vaccines provide the first hope for mitigating the devastating health and economic impacts resulting from coronavirus disease 2019 (COVID-19) and related public health orders. Recent case reports of reactions to COVID-19 vaccines have raised questions about their safety for use in individuals with allergies and those who are immunocompromised. In this document, we aim to address these concerns and provide guidance for allergists/immunologists.

**Methods:**

Scoping review of the literature regarding COVID-19 vaccination, adverse or allergic reactions, and immunocompromise from PubMed over the term of December 2020 to present date. We filtered our search with the terms “human” and “English” and limited the search to the relevant subject age range with the term “adult.” Reports resulting from these searches and relevant references cited in those reports were reviewed and cited on the basis of their relevance.

**Results:**

Assessment by an allergist is warranted in any individual with a suspected allergy to a COVID-19 vaccine or any of its components. Assessment by an allergist is NOT required for individuals with a history of unrelated allergies, including to allergies to foods, drugs, insect venom or environmental allergens. COVID-19 vaccines should be offered to immunocompromised patients if the benefit is deemed to outweigh any potential risks of vaccination.

**Interpretation:**

This review provides the first Canadian guidance regarding assessment of an adolescent and adult with a suspected allergy to one of the COVID-19 vaccines currently available, or any of their known allergenic components, and for patients who are immunocompromised who require vaccination for COVID-19. As information is updated this guidance will be updated accordingly.

## Introduction

Safe and effective vaccines provide the first hope for mitigating the devastating health and economic impacts resulting from coronavirus disease 2019 (COVID-19) and related public health orders. Both the Pfizer-BioNTech and Moderna products are currently approved in Canada, and further vaccines will likely become available in the coming months. A high rate of vaccine uptake across all sectors of Canadian society is a priority public health goal.

Recent case reports of reactions to COVID-19 vaccines have raised questions about their safety for use in individuals with allergies and those who are immunocompromised. In this document, we aim to address these concerns and provide guidance for allergists/immunologists. **This document is current as of January 10, 2021 and is based on available evidence to date.**

## Suggested approach to vaccination for allergists/immunologists in individuals with confirmed or suspected allergic contraindications to receiving COVID-19 vaccines

- **Assessment by an allergist is warranted in any individual with a suspected allergy to a COVID-19 vaccine or any of its components.** This includes anyone who has experienced a suspected allergic reaction after receiving the first dose of a COVID-19 vaccine, or someone with a suspected or confirmed allergy to a component of the vaccine. Proper assessment will help to clarify whether and how a COVID-19 vaccine can be (re)administered and, if necessary, can help in the selection of an alternative COVID-19 vaccine when one becomes available.
- **Assessment by an allergist is NOT required for individuals with a history of unrelated allergies, including to allergies to foods, drugs, insect venom or environmental allergens.** In these individuals, the available COVID-19 vaccines can be administered without any special precautions. As for the routine administration of all vaccines, they should be administered in a healthcare setting capable of managing anaphylaxis, and individuals should be observed for a minimum of 15–30 min following vaccination.
- These recommendations will be updated as appropriate.

### Summary

1. There is a low risk for allergic reactions associated with vaccines. Non-allergic reactions to vaccines are much more frequent than allergic reactions.
  - Vaccines activate the immune system, which will commonly result in minor side effects, including mild fever and local inflammatory reactions at the site of the injection.
  - Non-allergic reactions to vaccines also include anxiety-related adverse events that can mimic allergic reactions.
2. The nature and cause of the reactions to the Pfizer-BioNTech and Moderna COVID-19 vaccines remains unclear, including how many have been due to allergic reactions.
3. The feasibility of allergy testing for the COVID-19 vaccines is not yet known.
4. The Pfizer-BioNTech and Moderna COVID-19 vaccines contain polyethylene glycol (PEG), which has been identified as potentially allergenic, but it is not yet known whether allergy to PEG is responsible for the reported adverse reactions to these vaccines.
5. It is unknown whether allergy testing for PEG compounds will be relevant to the investigation of possible allergy to the Pfizer-BioNTech and Moderna COVID-19 vaccines.
6. Chlorhexidine hypersensitivity should be considered in relevant cases.
7. Graded administration of these vaccines in someone with a suspected or confirmed allergy to the vaccine or one of its components can be considered if further doses are required.

### There is a low risk for allergic reactions associated with vaccines. Non-allergic reactions to vaccines are much more frequent than allergic reactions

**Vaccines activate the immune system, which will commonly result in minor side effects, including mild fever and local inflammatory reactions at the site of the injection.** This may include redness, swelling, pain, and warmth at the injection sites [1]. These reactions are not a contraindication to receiving the same vaccine in the future, as they do not pose a risk for future allergic reactions to the vaccine.

**Non-allergic reactions to vaccines also include anxiety-related adverse events that can mimic allergic reactions,** and may include breath-holding, hyperventilation, and vasovagal syncope (fainting) (see Table 1 in the Canadian Immunization Guide: Anaphylaxis and other Acute Reactions following Vaccination) [2].

Acute localized allergic reactions at the site of the injection, consisting of urticaria and angioedema, are also possible, but the risk of systemic allergic reactions, including anaphylaxis, is considered extremely rare. Studies suggest that the estimated annual rate of anaphylaxis in Canada is approximately 0.4 to 1.8 cases per 1,000,000 doses of vaccine administration [2–4].

### The nature and cause of the reactions to the Pfizer-BioNTech and Moderna COVID-19 vaccines remains unclear, including how many have been due to allergic reactions

Recent publications suggest that the rate of anaphylaxis associated with the Pfizer-BioNTech vaccine may be approximately 10 times higher than the incidence reported with all previous vaccines”; [5, 6] however, we must be cautious not to repeat history. Previous experience with the pandemic H1N1 (pH1N1) vaccine has educated us that although the pH1N1 vaccine was initially reported to have caused a “rate of anaphylaxis 20 times greater than the historical average”, subsequent careful investigation revealed that a striking 96% of those initially reported to have experienced anaphylaxis after receiving the vaccine had no evidence of allergy to that vaccine [7].

Millions of doses of the Pfizer-BioNTech and Moderna COVID-19 vaccines have been safely administered around the world to date, with over 23 million vaccinated for COVID-19 worldwide to date [8] It is not yet known how many of the reported vaccine reactions to the Pfizer-BioNTech and Moderna COVID-19 vaccines are allergic in nature or what component of the vaccine those individuals may have reacted to. This remains under investigation.

### The feasibility of allergy testing for the COVID-19 vaccines is not yet known

The validity of epicutaneous and intradermal testing to the Pfizer-BioNTech and Moderna COVID-19 vaccines has not yet been established. In addition, the availability of the Pfizer-BioNTech and Moderna COVID-19 vaccines for the purpose of skin testing is not yet known.

### The Pfizer-BioNTech and Moderna COVID-19 vaccines contain polyethylene glycol (PEG), which has been identified as potentially allergenic, but it is not yet known whether allergy to PEG is responsible for the reported adverse reactions to these vaccines

Allergic reactions to vaccines can be elicited by the active vaccine component, or more commonly, by one of the other components [2–4].

Polyethylene glycol, commonly known as PEG, has been identified as the most likely potentially allergenic component of both Pfizer-BioNTech and Moderna COVID-19 vaccines, [9] though it is not yet known whether the PEG component of these vaccines is responsible for the reactions that have been reported to date. The other components of these vaccines, including the active mRNA components of both vaccines, are unlikely to be allergenic.

### It is unknown whether allergy testing for PEG compounds will be relevant to the investigation of possible allergy to the Pfizer-BioNTech and Moderna COVID-19 vaccines

Allergy to PEG has previously been reported. PEG compounds have a range of molecular weights, and allergic sensitization to PEG has mainly been documented for PEG with higher molecular weight and when present in higher concentration [10–15]. However, PEG is found in multiple products that are tolerated safely on a daily basis by many individuals in Canada, including bowel preparation products for surgical procedures, certain laxatives and other medications, certain skin care products and cosmetics, and some food and drinks.

A recent publication has suggested a possible role for allergy testing to PEG within the context of evaluation of allergy to these vaccines [16]. Although both epicutaneous and intradermal skin testing for PEG has been described within the context of case reports and research, [11–14] such testing has not been standardized and its validity is not well established. In addition, systemic reactions, including anaphylaxis, has been described as a result of both epicutaneous and intradermal testing to PEG [12, 14]. Furthermore, although cross-reactivity between different types of PEG has been suggested, [11, 12, 15] the Pfizer-BioNTech and Moderna COVID-19 vaccines contain different forms of PEG, and the degree of cross-reactivity between these PEG molecules has not yet been established. The Oxford-AstraZeneca COVID-19 vaccine has been approved by the United Kingdom (UK) and contains polysorbate 80 (which may cross-react with PEG), but the clinical implications of this are also unknown [16].

### Chlorhexidine hypersensitivity should be considered in relevant cases

Other exposures should also be considered as a possible source of adverse reactions during vaccination with these vaccines. Localized irritation and contact reactions have been described to compounds used to prepare the injection site. More specifically chlorhexidine, used to sterilize vaccine injection sites, may elicit allergic reactions. Skin testing for chlorhexidine allergy may be used to make a diagnosis but is not standardized [17].

### Graded administration of these vaccines in someone with a suspected or confirmed allergy to the vaccine or one of its components can be considered if further doses are required

**Fig. 1:**
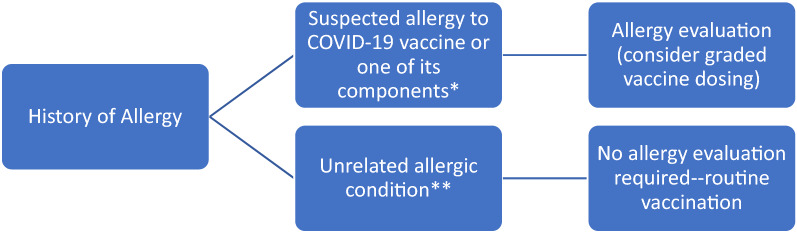
Algorithm of Evaluation in a Patient with a History of Allergy Prior to COVID-19 Vaccination. *Identified allergenic component of the COVID-19 vaccine is polyethylene glycol (PEG)**.** **Unrelated allergic conditions include food allergy (irrespective of severity), allergic rhinitis, asthma, eczema, stinging insect allergy

In summary, for a higher-risk patient who has previously experienced a suspected or confirmed severe allergic reaction to a COVID-19 vaccine or any of its components, allergy testing to the vaccine or its components is not required for the vast majority of these patients (Fig. 1). A reasonable and safe option for consideration as part of shared decision-making is the administration of the COVID-19 vaccine using a graded vaccine administration protocol. For higher-risk patients who are hesitant to proceed with graded vaccine administration, allergy testing remains an option after education that the predictive value of such testing is unknown. Allergy testing for lower-risk patients is NOT recommended to prevent delay in administration of COVID-19 vaccines.

Guidance for the cautious graded administration of a vaccine in someone with a confirmed IgE-mediated allergy to that vaccine or one of its components has previously been published: administer 0.05 mL 1:10 dilution, 10, 20, 30, and 40% of the full dose incrementally in alternate arms at 15 min intervals, followed by a minimum 30 min observation period. [see Table V in the referenced document] [18].

## Suggested approach to vaccination for allergists/immunologists in immunocompromised individuals

### COVID-19 vaccines should be offered to immunocompromised patients if the benefit is deemed to outweigh any potential risks of vaccination

Immunocompromised individuals are at high risk for severe COVID-19 and should be considered a priority group for intervention that will reduce their risk of this disease.

The Pfizer-BioNTech and Moderna COVID-19 vaccines are mRNA vaccines, and as such are not live vaccines and can be administered to immunocompromised individuals. However, it is not yet known how immunocompromised individuals will tolerate or respond to the COVID-19 vaccines, as there are no data yet available in these groups. The Canadian product monographs for both the Pfizer-BioNTech and Moderna vaccines state: “Immunocompromised persons, including individuals receiving immunosuppressant therapy, may have a diminished immune response to the vaccine” [19, 20].

The National Advisory Committee on Immunization (NACI) currently recommends that “COVID-19 vaccine should not be routinely offered to individuals who are immunosuppressed due to disease or treatment until further evidence is available” However, they further state that these vaccines “may be offered… in this population if a risk assessment deems that the benefits outweigh the potential risks for the individual” [9].

In the UK, immunosuppressed individuals are considered a priority group to receive COVID-19 vaccines (see Table 3 in the referenced document) [21]. The European Society for Immunodeficiency (ESID) currently recommends that “patients with PID receive COVID-19 vaccinations provided that they are not live vaccines” [22]. The European League Against Rheumatism (EULAR) recommends that immunocompromised patients be vaccinated against COVID-19 [23].

Accordingly, the CSACI suggests that COVID-19 vaccines should be offered to immunocompromised patients following a careful risk assessment if the benefit is deemed to outweigh any potential risks of vaccination. We recognize that this is a rapidly evolving area and will be following this closely, with updates made to this recommend as necessary.

## Data Availability

Not applicable.

## Abbreviations

CSACI: Canadian Society of Allergy and Clinical Immunology
COVID-19: Coronavirus disease 2019
pH1N1: Pandemic H1N1
UK: United Kingdom
PEG: Polyethylene glycol
NACI: National Advisory Committee on Immunization
ESID: European Society for Immunodeficiency
EULAR: European League Against Rheumatism
